# Financial incentives to improve adherence: more clarity about their purpose may help the debate

**DOI:** 10.1192/bjb.2023.4

**Published:** 2023-06

**Authors:** Stefan Priebe

**Affiliations:** Queen Mary University of London, London, UK

**Keywords:** Financial incentives, adherence, medication, mental illness, psychiatry

## Abstract

Financial incentives for medication adherence have been controversial in mental healthcare. Much of the debate, however, may be based on a misconception of what financial incentives are and what their purpose is. Financial incentives are not meant to influence informed consent about treatment decisions, but to bridge the gap between intentions and behaviour and help patients achieve adherence to a treatment that they have agreed to. In this context, patients’ positive views may reflect that the use of financial incentives can support a good therapeutic relationship rather than undermine it.

Financial means are widely used to influence our health behaviour. Mostly, these take the form of penalising us when we do something that may have a negative impact on our health and/or the health of others. Taxes on alcohol and cigarettes are intended to make us drink and smoke less, and the congestion charge should encourage us not to drive into central London. Financial incentives are no penalisation, but something additional to what one normally gets, such as a prize for doing well at sport or in education.

Financial incentives have been used to improve adherence to treatments across medicine.^1^ When used in specialties outside psychiatry, the discussion usually focuses on their effectiveness.^2^ In psychiatry, however, where incentives have been mainly used to influence adherence to antipsychotic medication, they have been fiercely debated for a number of reasons. Hodson and colleagues summarise some of those reasons.^3^ Their article also makes a point that so far has rarely featured in the discussion: patients view financial incentives as positive and are in favour of them. One might think that this is a trivial statement, since most people would like to get more money if they can. However, the point is actually very important.

Before explaining this further, it may help to clarify what financial incentives to improve treatment adherence are and what their exact their purpose is.

## The purpose of financial incentives in healthcare

Financial incentives are not meant to influence the treatment decision-making of a patient. If a patient considers and balances the pros and cons of a given medication or other treatment, financial incentives should not tip their preference towards accepting the treatment. Most people would regard it as unacceptable if clinicians tried to override patients’ legitimate and proper concerns about a given treatment by offering money. Such offers may put the poor at particular risk as they might not be able to afford to reject the incentive and therefore accept a potentially harmful treatment. They might also open the doors to legal challenges if the treatment fails and the patient suffers serious side-effects.

The purpose of incentives is to help patients achieve adherence to a treatment to which they have given informed consent. Of course, informed consent is an artificially simplified and categorical concept that leaves little room for grey zones and ambivalence. Still, once patients have provided informed consent, clinicians need to accept that. So, why do patients not just adhere to something that they have agreed to doing?

Most of us have good intentions that we do not always carry out. We do things that we would prefer not to do, such as drinking too many glasses of wine or spending more time in front of the computer screen than we feel is good for us and our social life. And we fail to do other things that we strongly feel we should be doing, such as taking more physical exercise or being nicer to our partners. This brief editorial is not the place to consider psychological theories for why we are not always doing what we believe we should be doing and what is good for us. Suffice to conclude that life is complex and that discrepancies between intentions and behaviour can exist. The Gospel according to Matthew (26:41) in the Bible already refers to this dilemma: ‘the spirit is willing, but the flesh is weak’. Accordingly, rates of non-adherence to medication tend to be about 40–50% among people with chronic conditions such as diabetes and hypertension.^4^

How can we help patients to achieve what they – in principle – have agreed to? This is where financial incentives come into play, and this why they are – as Hodson and colleagues explain^3^ – distinct from payments. People are paid to do something for someone else – in contrast, incentives should help people achieve something in their own interest. A good example of their use in healthcare is smoking cessation. Financial incentives are not used to persuade someone to quit smoking who is determined to continue. They have, however, been used to help those who want to quit to achieve their intentions, and in such a situation, they can be effective.^2^

## The evidence base in mental healthcare: the FIAT study

In mental healthcare, financial incentives have been shown to be beneficial in terms of increasing treatment adherence. In the FIAT study, we found that incentives halved the non-adherence to antipsychotic maintenance medication.^5^ The difference became apparent once the incentives had been started, lasted for the 1-year period during which incentives were provided and disappeared after the incentives were stopped.^6^ Thus, financial incentives had some positive effect without being a miracle tool.

When the findings of the FIAT study were disseminated, many clinicians expressed concerns. Some of the concerns speculated about patients’ feelings and argued that patients would not feel respected and valued when they were offered money to take the medication. The article by Hodson and colleagues rejects this assumption and shows that most patients do not have a problem with the idea of financial incentives; on the contrary most express positive views about it.^3^ So, why are many clinicians still so opposed^7^?

## Do financial incentives really risk the therapeutic relationship?

As it is impossible in this brief editorial to discuss all potential reasons for rejecting financial incentives, I will address only two out of the list that Hodson and colleagues have provided. One is the concern that patients may use the money to buy illegal drugs, which seems an outdated patronising view. Clinicians can hardly aim to keep patients poor just to prevent them from buying illegal drugs. A more valid point is that clinicians might feel that their own trust-building skills and their focus on establishing helpful relationships may be devalued. For clinicians, it may indeed feel disappointing if offering an incentive of only £15 (which was the incentive in the FIAT study for adherence to one depot medication) can have a more positive impact on patient behaviour than all the communication and relationship-building skills that we have acquired in many years of training and practice. On the other hand, some clinicians with experience of offering financial incentives appreciate the possibility of offering something positive to patients for good adherence instead of consistently outlining and emphasising the unpleasant risks of poor adherence.^8^ Hodson and colleague's point suggests that – at least with some patients – offering financial incentives might be positive for the therapeutic relationship rather than undermine it.

## About the author

**Stefan Priebe** is Professor for Social and Community Psychiatry, heading the Unit for Social and Community Psychiatry, Queen Mary University of London, London, UK.

## Data Availability

Data availability is not applicable to this article as no new data were created or analysed in this study.
